# Plasmid-mediated quinolone resistance genes detected in Ciprofloxacin non-susceptible *Escherichia coli* and *Klebsiella* isolated from children under five years at hospital discharge, Kenya

**DOI:** 10.1186/s12866-023-02849-2

**Published:** 2023-05-13

**Authors:** Kevin Kariuki, Mame Mareme Diakhate, Susan Musembi, Stephanie N. Tornberg-Belanger, Doreen Rwigi, Timothy Mutuma, Elizabeth Mutuku, Kirkby D. Tickell, Olusegun O. Soge, Benson O. Singa, Judd L. Walson, Patricia B. Pavlinac, Samuel Kariuki

**Affiliations:** 1 Centre for Microbiology Research, Kenya Medical Research Institute (KEMRI), Nairobi, Kenya; 2 Department of Biochemistry, Microbiology and Biotechnology, Kenyatta University, Nairobi, Kenya; 3 Department of Global Health, University of Washington, Seattle, WA USA; 4 Internal Medicine, (Infectious Diseases), Atrium Health Wake Forest Baptist Health, Winston-Salem, NC USA; 5 Centre for Clinical Research, Kenya Medical Research Institute (KEMRI), Nairobi, Kenya; 6 Department of Epidemiology, University of Washington, Seattle, WA USA; 7 Department of Pediatrics and Medicine (Allergy and Infectious Diseases), University of Washington, Seattle, WA USA

**Keywords:** Ciprofloxacin, *Escherichia coli*, *Klebsiella* spp, Post-hospital discharge, Fluoroquinolones, Antimicrobial resistance

## Abstract

**Background:**

The increasing spread of fluoroquinolone resistant enteric bacteria is a global public health concern. Children recently discharged from the hospital are at high risk of carriage of antimicrobial resistance (AMR) due to frequent exposure to antimicrobials during inpatient stays. This study aimed to determine the prevalence, correlates of ciprofloxacin (CIP) non-susceptibility, and distribution of plasmid-mediated quinolone resistance (PMQR) genes in *Escherichia coli (E. coli)* and *Klebsiella* spp isolated from children under five years being discharged from two Kenyan Hospitals.

**Methods:**

*E. coli* and *Klebsiella* spp were isolated from fecal samples from children discharged from hospital and subjected to antimicrobial susceptibility testing (AST) by disc diffusion and E-test. CIP non-susceptible isolates were screened for seven PMQR genes using multiplex polymerase chain reaction (PCR). Poisson regression was used to determine the association between the carriage of CIP non-susceptible isolates and patient characteristics.

**Results:**

Of the 280 CIP non-susceptible isolates: 188 *E. coli* and 92 *Klebsiella* spp isolates identified among 266 discharged children, 195 (68%) were CIP-non-susceptible with minimum inhibitory concentrations (MICs) of ≥ 1 µg/mL. Among these 195 isolates, 130 (67%) had high-level CIP MIC =  ≥ 32 µg/mL). Over 80% of the isolates had at least one PMQR gene identified: *aac(6’)lb-cr (60%), qnrB (24%)*, *oqxAB (22%), qnrS (16%),* and *qepA (6%),* however, *qnrA* was not identified in any isolates tested. Co-carriage of *qnrB* with *acc(6’)-lb-cr* was the most predominant accounting for 20% of all the isolates. Ceftriaxone use during hospital admission and the presence of extended spectrum beta-lactamase (ESBL) production were significantly associated with the carriage of CIP non-susceptible *E. coli* and *Klebsiella* spp.

**Conclusion:**

CIP non-susceptibility is common among *E. coli* and *Klebsiella* spp isolated from hospital discharged children in Kenya. Carriage and co-carriage of PMQR, including the newly identified *qepA* gene, were frequently observed. These findings suggest that children leaving the hospital may serve as an important reservoir for transmission of resistant *E. coli* and *Klebsiella* spp to the community. Enhanced surveillance for AMR determinants is critical to inform interventions to control antimicrobial-resistant bacteria.

**Supplementary Information:**

The online version contains supplementary material available at 10.1186/s12866-023-02849-2.

## Background

AMR is a global public health threat associated with morbidity, rehospitalization, longer hospital stays, and mortality [1, 2]. In sub-Saharan Africa (SSA), antibiotic-resistant pathogens are major drivers of morbidity and mortality in children under five years of age, fuelled by inappropriate antibiotic use and poor sanitation [3] leading to the selection and spread of antibiotic-resistant bacteria [4]. Estimates by the 2019 report on the Global Burden of Bacterial AMR identified SSA as the highest contributor to the global AMR burden [5].

Commensal resident gut bacteria such as *E. coli* and *Klebsiella pneumoniae* (*K. pneumoniae*) play a critical role in AMR as they act as reservoirs for the carriage of AMR determinants [6, 7]. These enteric bacteria can become pathogenic or may transfer AMR genes to other pathogenic Enterobacterales, such as *Salmonella* and *Shigella* [8]. The acquisition of AMR and virulence factors by commensal *E. coli* and *Klebsiella* spp is mediated by mobile genetic elements, such as plasmids and transposons, via horizontal gene transfer [9]. Antibiotic resistance in commensal enteric bacteria, such as *K. pneumoniae* and *E. coli*, has been reported in 85–90% of World Health Organization (WHO) member state regions [10].

Fluoroquinolones such as ciprofloxacin (CIP) are effective broad-spectrum antibiotics used for the treatment of bacterial infections making them a recommended choice of therapy for enteric infections such as salmonellosis and shigellosis [11]. The emergence of fluoroquinolone resistance has reduced therapeutic options, especially for Enterobacterales infections [12]. Fluoroquinolone resistance is mediated by two mechanisms: chromosomal mutations in DNA gyrase and topoisomerase IV enzymes and plasmid-mediated quinolone resistance (PMQR). Mutations in fluoroquinolone binding sites during DNA replication mediate high-level fluoroquinolone resistance[13]. Mechanisms of PMQR genes include protection of DNA gyrase and topoisomerase IV from quinolone activity mediated by *qnr* genes: *qnrA*, *qnrB*, *qnrC*, *qnrD,* and *qnrVC* [14]*.* The aminoglycoside-modifying enzyme encoded by *aac(6’)-Ib-cr* is involved in the acetylation of fluoroquinolones leading to reduced susceptibility to CIP and norfloxacin [14]. The final mechanism is enhanced efflux pump activity mediated by quinolone efflux pump *(qepA)* and *oqxAB* associated with reduced susceptibility to fluoroquinolone and increased ESBLs [15].

There is a paucity of data available on fluoroquinolone resistance and PMQR determinants in commensal bacteria, especially in children under five years in Kenya. In this study, we sought to determine the prevalence and the distribution of PMQR determinants mediating CIP non-susceptibility in *E. coli* and *Klebsiella* spp isolates from children being discharged from the hospital. In addition, we identified correlates of carriage of CIP non-susceptible isolates. This in-depth analysis will help inform the burden of CIP resistance carriage in children under five years being discharged from hospitals in SSA.

## Results

### Participant population and baseline characteristics

**Fig. 1:**
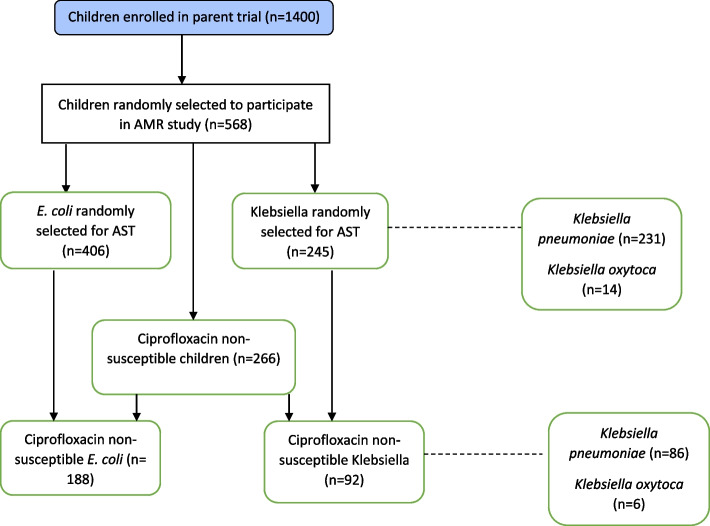
Flowchart of participants isolates. AMR = Antimicrobial resitanace; AST = Antimicrobial susceptiability testing; CIP = Ciprofloxacin; *E. coli* = *Escherichia coli*; *n* = Number

Six hundred and fifty-one isolates (406 *E. coli* and 245 *Klebsiella* spp) were isolated from 568 children and subjected to AST (Fig. 1). Among the 568 children, 343 (60.4%) were discharged from Kisii Teaching and Referral Hospital (KTRH) and 225 (39.6%) were discharged from Homabay County Referral Hospital (HCRH), 348 (61.3%) were less than two years of age and 230 (40.5%) were female (Table 1). Prior to discharge, the median duration of hospitalization was 3 days [interquartile range (IQR) 2,6 days] with 203 (35.7%) children being hospitalized for ≥ 4 days. The majority of the children (97.7%) had their human immunodeficiency virus (HIV) status determined with 12 (2.1%) being HIV-infected while 70 (12.3%) were HIV-exposed. There were more HIV-exposed children in HCRH 51 (22.7%) compared to KTRH 19, (5.5%). Common diagnoses at discharge were pneumonia 164 (28.9%), malaria 155 (27.3%), and diarrhoea 108 (19%) (Table 1). The majority of the children 502 (88.4%) had an antibiotic prescribed during their hospitalization with penicillins 359 (63.2%) being the most prescribed antibiotic followed by gentamicin 316 (55.6%) and ceftriaxone 187 (32.9%). Fluoroquinolones were rarely administered during hospitalization with CIP being prescribed to only 2 (0.9%) children in HCRH and none among children from KTRH. Children at KTRH 328 (95.6%) compared to HCRH 174, (77.3%) were more likely to be prescribed an antibiotic. Nearly half of the children, 266 (46.8%) had CIP non-susceptible isolates (46.3% *E. coli* and 37.6% *Klebsiella* spp). Of the 83 children who had both *E. coli* and *Klebsiella* spp isolated, 14 (2.46%) had CIP non-susceptibility in both isolates.

**Table 1:** Participant baseline characteristics

	**Kisii**		**Homa Bay**		**Total**	
***n***	**N:343**		**N:225**		**N:568**	
**Sociodemographic**						
** Age(months)**						
1 to 5	53	15.5%	20	8.9%	73	12.9%
6 to 11	73	21.3%	44	19.6%	117	20.6%
12 to 23	87	25.4%	71	31.6%	158	27.8%
24 to 59	130	37.9%	90	40%	220	38.7%
** Sex**						
Male	205	59.8%	133	59.1%	338	59.5%
Female	138	40.2%	92	40.9%	230	40.5%
** Duration of Hospitalization(days)**						
0 to 2	100	29.2%	66	29.3%	166	29.2%
3 to 4	121	35.3%	73	32.4%	194	34.2%
> 4	117	34.1%	86	38.2%	203	35.7%
Unknown^a^	5	1.5%	0	0	5	0.9%
Median (25%, 75%)	3	(2, 6)	4	(2, 6)	3	(2, 6)
** HIV Status**						
HIV unexposed	309	90.1%	164	72.9%	473	83.3%
HIV exposed, uninfected	19	5.5%	51	22.7%	70	12.3%
HIV infected	6	1.7%	6	2.7%	12	2.1%
HIV-uninfected/exposure status unknown	9	2.6%	4	1.8%	13	2.3%
** Diagnosis at Discharge**^b,c^						
Diarrhoea	65	19%	43	19.1%	108	19%
Lower respiratory tract infection	117	34.1%	47	20.9%	164	28.9%
Malaria	68	19.8%	87	38.7%	155	27.3%
Malnutrition	24	7%	18	8%	42	7.4%
Pneumonia	117	34.1%	47	20.9%	164	28.9%
Upper respiratory tract infection	36	10.5%	10	4.4%	46	8.1%
** Any antibiotic used during admission (enrollment visit)**						
Any antibiotics used	328	95.6%	174	77.3%	502	88.4%
Azithromycin	2	0.6%	2	0.9%	4	0.7%
Ceftriaxone	102	29.7%	85	37.8%	187	32.9%
Penicillin	268	78.1%	91	40.4%	359	63.2%
Gentamicin	241	70.3%	75	33.3%	316	55.6%
Ciprofloxacin	0	0	2	0.9%	2	0.4%

^a^Missing either admission or discharge dates (*n* = 5)

^b^Diagnosis are not mutually exclusive

^c^No documented diagnosis at discharge (*n* = 23)

### Correlates for carriage of fluoroquinolone non-susceptible *E. coli* or *Klebsiella* at hospital discharge

Children who received an antibiotic during hospitalization were 69% more likely to have a CIP non-susceptible *E. coli* isolate (PR 1.69, [95%CI = 1.09, 2.63], *p* = 0.01) and over two times more likely to have a CIP non-susceptible *Klebsiella* spp isolate (PR 2.61, [95%CI = 1.05, 6.53], *p* = 0.01). The presence of ESBL carriage was also associated with the presence of either a CIP non-susceptible *E. coli* or *Klebsiella* spp isolate. Length of hospital stay was associated with CIP non-susceptible *E. coli* and children with hospitalizations extending more than 4 days were nearly 40% more likely to have CIP non-susceptible *E. coli* (PR 1.38 [95%CI 1.07, 1.78] *p* = 0.01). The use of either CIP or ceftriaxone were equally associated with CIP non-susceptible *Klebsiella* or *E. coli*. Children hospitalized for diarrhoea were 27% less likely to have a CIP non-susceptible *E. coli* compared to those who did not present with diarrhoea (PR 0.73 [95%CI 0.53, 1.0] *p* = 0.03). Similar magnitudes of association for hospital length and diarrhoea diagnosis were observed in *Klebsiella* isolates but were not statistically significant (Table S1).

### Distribution of CIP non-susceptible isolates

Among the 266 children with CIP non-susceptible isolates, we isolated 280 CIP non-susceptible isolates: 188 and 92 *E. coli* and *Klebsiella* spp, respectively. Of the 266 children, 14 had both a CIP non-susceptible *E. coli* and *Klebsiella* spp isolated. Among the 92 *Klebsiella* spp, 86 were *K. pneumoniae* and 6 were *Klebsiella oxytoca (K. oxytoca)* (Fig. 1).

CIP MICs values for the 280 CIP non-susceptible isolates ranged between 0.25 – 32 µg/mL with CIP MIC_50_ (µg/mL) of 32 µg/mL. Of the 280 isolates, 214 (76.4%) were resistant while 61 (21.8%) were intermediate. Among the resistant isolates, high-level CIP resistance (MIC ≥ 32 µg/mL) was common in almost half of the isolates (46.4%) most commonly among *E. coli* 102 (54.3%) and slightly less in *Klebsiella* spp (28/92, 30.4%). The CIP MIC distribution, MIC_50_ and MIC_90_ among the isolates are shown in Table 2.

**Table 2:** Distribution of MIC (µg/mL) per organism

	**Frequency of isolates with indicated MIC value**								**CIP MIC**_**50**_ (µg/mL)	**CIP MIC**_**90**_(µg/mL)
MIC values (µg/mL)	**0.25**	**0.5**	**1**	**2**	**4**	**8**	**16**	**32**		
Bacterial species										
*E. coli*	2	51	9	7	10	2	5	102	32	32
*K. oxytoca*	1	0	0	0	0	1	0	4	32	32
*K. pneumoniae*	1	10	23	23	2	1	2	24	2	32
Number. of occurrences(%)	4 (**1.4**)	61 **(21.8)**	32 (**11.4**)	30 (**10.7**)	12 (**4.3**)	4 (**1.4**)	7 (**2.5**)	130 (**46.4**)		

*CIP* Ciprofloxacin, *E. coli Escherichia coli*, *K. oxytoca Klebsiella oxytoca*, *K. pneumoniae Klebsiella pneumoniae*, *MIC* Minimum inhibitory concentration, *MIC*_*50*_ MIC value at which > 50% of the isolates are inhibited, *MIC*_*90*_ MIC value at which ≥ 90% of the isolates are inhibited

### Distribution of PMQR determinants

Six different PMQR determinants: *qnr* (*qnrB*, and *qnrS*), enzyme modifying *aac(6′)-Ib-cr,* and efflux pumps *(qepA, oqxA,* and *oqxB*) were detected (Table 3). At least one of the PMQR genes was detected in nearly all (224/280, 80%) of the screened isolates. Most *E. coli* and *Klebsiella* spp (40%) isolates had at least one *qnr* determinant detected. Of the *qnr* genes, *qnrB* was the most commonly detected *qnr* gene (20/188, 11%) and 47/92(51%) in *E. coli* and *Klebsiella* spp, respectively. In *E. coli*, *qnrS* was the most detected *qnr* gene (27/188, 14%). The *qnrA* gene was not detected in any of the *E. coli* or *Klebsiella* spp isolates.

**Table 3:** Distribution of PMQR determinants per organism

	*E. coli*	*K. oxytoca*	*K. pneumoniae*	Total
PMQR genes	*N* = 188	*N* = 6	*N* = 86	*N* = 280
*qnrB*	20 (11%)	2 (33%)	45 (52%)	67 (24%)
*qnrS*	27 (14%)	1 (17%)	18 (21%)	46 (16%)
*aac(6′)-Ib-cr*	89 (47%)	5 (83%)	73 (85%)	167 (59%)
*qepA*	16 (9%)	0 (0%)	0 (0%)	16 (6%)
*OqxA*	2 (1%)	4 (67%)	83 (97%)	89 (32%)
*OqxB*	0 (0%)	3 (50%)	59 (69%)	62 (22%)
*OqxAB*	0 (0%)	3 (50%)	58 (67%)	61 (22%)

*E. coli Escherichia coli*, *K. oxytoca Klebsiella oxytoca*, *K. pneumoniae Klebsiella pneumoniae*, *N* Number, *PMQR* Plasmid mediated quinolone resistance

The most predominant PMQR gene was *aac(6’)-lb* (167/280, 59%) identified in more than half of all CIP non-susceptible isolates. *Klebsiella* spp had more *aac(6’)-lb* positive isolates with (78/89, 85%) *Klebsiella* compared to (89/188, 47%) of *E. coli* isolates. All isolates carrying the *aac(6’)-lb* gene were positive for the *cr* variant. The *qepA* gene was only detected in (16/188, 9%) *E. coli* CIP non-susceptible isolates. The o*qxAB* complex was the most dominant efflux pump detected (61/92, 66.3%) in *Klebsiella*, however, none were detected in *E. coli*. Only (2/188, 1%) *E. coli* isolates had the *oqxA* gene, however, both of these isolates lacked the *oqxB* gene therefore none of the *E. coli* isolates carried the *oqxAB* complex.

### *QepA* sequence analysis

DNA sequencing of the *qepA* gene from 16 *E. coli* isolates revealed amino acid substitutions at codons 95 and 134. Double amino acid substitutions F95L and V134I were common in 9/16 (56.3%) *E. coli* isolates. Six *qepA* positive *E. coli* isolates carried had no amino acid substitution while one isolate had only V134I amino acid substitution (Table 4). Assigned accession numbers for the *qepA* gene from 8 representative isolates submitted to GenBank are as follows: OP918677, and OQ031499-OQ031505.

**Table 4:** MICs and amino acid changes in *qepA E. coli* isolates

Isolate ID	Accession Number	Site	Organism	CIP MIC	INTER	*qepA* Variants	Amino acid variation	
							**F95**	**V134**
**E24**	OQ031502	Kisii	*E. coli*	32	R	*-*	F95	V134I
**E28**	OQ031503	Kisii	*E. coli*	2	R	*qepA1*	F95	V134
**E40**	OQ031504	Kisii	*E. coli*	2	R	*qepA1*	F95	V134
**E42**	OQ031505	Kisii	*E. coli*	32	R	*qepA1*	F95	V134
**E49**	OQ031499	Homabay	*E. coli*	32	R	*qepA4*	F95L	V134I
**E66**	OP918677	Kisii	*E. coli*	32	R	*qepA4*	F95L	V134I
**E79**	OQ031500	Homabay	*E. coli*	32	R	*qepA4*	F95L	V134I
**E80**	OQ031501	Homabay	*E. coli*	32	R	*qepA4*	F95L	V134I

*CIP* Ciprofloxacin, *INTER* Interpretation of susceptibility to Ciprofloxacin according to CLSI guidelines 2020 [16], *Isolate ID* Isolate identification number, *MIC* Minimum inhibitory concentration, *qepA* quinolone efflux pump

### Distribution of co-carriage of PMQR determinants per organism

A total of 225/280 (80%) isolates had at least one PMQR gene including; all 92 *Klebsiella* spp and most *E. coli* 133/189 (70.37%). Interestingly, *qnrB* and *qnrS* co-occurred in two *K. pneumoniae* (0.71%) isolates. Co-carriage of *qnrB* with *acc (6’) lb-cr* was present in 12/188 (6.4%) *E. coli* and *aac(6′)-Ib-cr* with *oqxAB* detected in 47/92 (51%) *Klebsiella* spp were the most prevalent combination of PMQR gene combinations. *E. coli* isolates had three notable combinations of different PMQR determinants with *qnr* combination being predominant in the co-existence of genes. On the other hand, *Klebsiella* spp had as many as nine different combinations and similarly, *qnr* gene combinations were predominant in the different combinations of determinants. The most common co-carriage in both bacterial species was *qnrB* with *acc (6’) lb-cr* found in 56/280 (20%). Additionally, *acc(6’)-lb-cr* co-existed with a majority of PMQR genes in both *E. coli* and *Klebsiella* spp isolates (Table 5).

**Table 5:** Co-carriage of PMQR determinants per organism

	Organism		
PMQR genes present	*E. coli*	*Klebsiella* spp	No. of occurrences
At least one PMQR	133/188	92/92	225/280
*qnrB* + *qnrS*	0	2	2
*qnrB* + *qnrS* + *aac(6′)-Ib-cr*	0	1	1
*qnrB* + *qnrS* + *aac(6′)-Ib-cr* + *oqxAB*	0	1	1
*qnrB* + *aac(6′)-Ib-cr*	12	44	56
*qnrB* + *acc (6’) lb-cr* + *oqxAB*	0	25	25
*qnrB* + *oqxAB*	0	13	13
*aac(6′)-Ib-cr* + *oqxAB*	0	47	47
*qnrS* + *aac(6′)-Ib-cr*	6	15	21
*qnrS* + *aac(6′)-Ib-cr* + *oqxAB*	0	10	10
*qepA* + *qnrS*	3	0	3
*qepA* + *aac(6′)-Ib-cr*	3	0	3

*E. coli Escherichia coli*, *No.* Number, *PMQR* Plasmid mediated quinolone resistance, *spp* species

## Discussion

Fluoroquinolone resistance in Enterobacterales in children under five years recently discharged from the hospital is of great public health concern due to the risk of transmission of these bacteria to the community, and treatment failure, which may require re-hospitalization during the post-hospital discharge period. This study sought to determine the prevalence and correlates of CIP non-susceptibility, and distribution of PMQR genes in *E. coli* and *Klebsiella* spp*,* isolated from children under five years recently discharged from hospitals. We observed a high level of CIP resistance among children being discharged from two hospitals in western Kenya and multiple fluoroquinolone resistance genes in *Klebsiella* spp and *E. coli*. Our findings show high levels of MICs to CIP in a majority of the CIP non-susceptible isolates, which is disturbing due to the relationship between increasing MICs leading to fluoroquinolone non-susceptibility and fluoroquinolone treatment failure [17]. This is particularly important given that fluoroquinolones are recommended therapies for the treatment of enteric infections such as shigellosis and salmonellosis [18, 19].

CIP non-susceptibility was detected in commensal *E. coli* (46%) and *Klebsiella* spp (38%) isolated from children being discharged from hospital and there was concomitant high carriage of PMQR genes (80%) among the isolates. This is despite less than 1% of the hospitalized children receiving fluoroquinolone antibiotics during hospitalization. The high fluoroquinolone non-susceptibility observed could be attributed to co-selection pressure mediated by non-fluoroquinolone antibiotics especially cephalosporins such as ceftriaxone, facilitating the selection and carriage of PMQR genes as previously reported [20]. This situation is further exacerbated by resistance pressure mediated by ESBL production which is evident in this study; the presence of ESBL production is highly associated with CIP non-susceptibility carriage [21]. Almost all children in the study population received an antibiotic during their in-patient stay with penicillin, gentamicin, and ceftriaxone being the most prescribed antibiotics consistent with other findings in Kenya [22]. Antimicrobial usage has been associated with selective pressure for AMR in gut bacteria [23]; our findings show that there is a strong correlation between antibiotic use and the carriage of CIP non-susceptible bacteria. Notably, children presenting with diarrhoea were less likely to carry a CIP non-susceptible *E. coli.* Children with diarrhoea may be less likely to be treated with an antibiotic [24], treated with fewer antibiotics, and/or treated with shorter courses of antibiotics compared to sepsis, malnutrition and pneumonia. Despite being the guideline-indicated antibiotic, CIP is rarely used for diarrhoea in this setting due to lack of availability and cost. Another reason could be during diarrhoeal episodes, there is gut dysbiosis that alters the gut composition [25] including resistant bacteria colonizing the gut. Thereby children presenting with diarrhoea would have a lower chance of carrying CIP non-susceptible *E. coli* compared to children without diarrhoea who have had the gut intact with colonized resistant bacteria.

PMQR genes facilitate low-level fluoroquinolone resistance, however, they select for higher-level resistance mediated by mutations on genes encoding gyrase and topoisomerase enzymes [13]. In this study, we detected six (*qnrB*, *qnrS, aac(6’)lb-cr, qepA, oqxA,* and *oqxB*) PMQR genes mediating fluoroquinolone resistance in both *E. coli* and *Klebsiella* spp. One of the six PMQR genes (*qepA*) had not previously been detected in clinical isolates in Kenya. The *qepA* gene has been reported in very few studies within the SSA region: Chad, Malawi, Egypt, Sierra Leon, and Nigeria [26–30]. Other PMQR determinants that have been identified in Kenya are *aac(6’)-lb-cr*, *qnrB*, *qnrS* in *E. coli* [31], *qnrS,* and *oqxAB* in *K.pneumoniae* [32, 33]. Intestinal carriage of PMQR genes in these bacteria has been reported in several studies in SSA and globally [34–36]. This is particularly worrying due to their potential transfer of these genetic determinants through mobile genetic elements to pathogenic bacterial species, thereby mediating the transmission of resistant bacteria that may result in treatment failure.

The *aac(6′)-Ib-cr* gene was the most commonly detected PMQR determinant. Most of the *Klebsiella* spp (84.78%) harboured the gene aminoglycoside modifying enzyme while almost half of the *E. coli* isolates (90/188, 48%) harbored the gene which is consistent with findings from previous studies [37]. The aminoglycoside acetyltransferase enzymes have not only been associated with reduced susceptibility to fluoroquinolones but also to aminoglycosides, thus limiting effective antibiotic treatment [38]. *Qnr* genes were the second most widely detected PMQR determinants associated with resistance to fluoroquinolones with prevalence rates of 40%. The prevalence of *qnr* genes (*40*%) was higher compared to previous studies from Kenya which reported (2%) and (8.4%) [33, 39]. The *qepA* gene*,* one of the most recently identified PMQR determinants, has been associated with decreased susceptibility to fluoroquinolones and increased MIC levels [13]. This determinant was detected in (16/188, 8.5%) *E. coli* isolates which was slightly lower compared to previous studies in Nigeria (18.5%) and Sierra Leon (23%) [28, 30]. DNA sequencing confirmed the existence of the *qepA* gene among *E. coli* isolates in Kenya with F95L and V134I amino acid substitution consistent with amino acid substitution reported in the *qepA4* allele [40]. To our knowledge, this is the first report of the detection of *qepA* in Kenya; this is worrisome for public health and calls for more active fluoroquinolone resistance surveillance.

Co-carriage of PMQR plays a critical role in multidrug resistance as it influences increased MICs leading to decreased susceptibility to fluoroquinolone antibiotics that may lead to treatment failure. We observed high multiple co-carriage in both *E. coli* and *Klebsiella* fluoroquinolone non-susceptible isolates with co-existence of *aac(6’)lb-cr* and *qnrB*, or *qnrS* genes, being the most predominant co-carriage in both bacteria. The prevalence of co-carriage between *qnrB* and *aac(6’) lb-cr* (20%) was found to be higher compared to findings from previous studies in SSA [41]. We observed co-carriage of *qnrB* with *qnrS* in two *K. pneumoniae* isolates, a phenomenon that has previously been reported in *Klebsiella* spp, however, its prevalence in this study was much lower compared than 18.75% reported in Togo [21]. This co-carriage could be attributed to multiple plasmids carrying the different *qnr* genes within the same genetic environment as has been previously demonstrated [42]. Notably, 12 isolates with the rare *qepA* co-existed with other PMQR genes (*qnrS* or *aac(6’)lb-cr*) which was consistent with findings from other previous studies [43]. Interestingly, one *K. pneumoniae* isolate co-harboured all determinants detected in this study except *qnrA* and *qepA.* This is concerning as the co-existence of multiple PMQR genes has been linked to resistance to multiple antibiotic classes due to the carriage of multiple plasmids carrying resistance determinants to other classes of antibiotics.

This is one of the few studies that has characterized AMR determinants in children post-hospital discharge in SSA settings, including screening for a wide range of PMQR genetic determinants, highlighting the greater diversity and distribution of fluoroquinolone resistance genes. In addition, the focus on commensal *E. coli* and *Klebsiella* spp*,* two commonly isolated Enterobacterales associated with the carriage of AMR determinants as indicator organisms for AMR carriage rather than pathogenic bacteria was important due to their ability to transfer AMR genetic elements.

This study had some limitations. CIP non-susceptibility was determined in a subset of isolates from children after discharge, which means there could be more non-susceptible isolates that were not screened. Only two children in this study received CIP or rather a fluoroquinolone during admission, this may not be sufficient to clearly show the role of fluoroquinolone resistance in poor patient outcomes during the post-discharge period. Limiting the analysis to PCR detection only, other mechanisms mediating resistance such as point mutations which could be detected by comprehensive whole-genome sequencing analyses were not captured. Being a cross-sectional study at the point of hospital discharge, we were unable to determine whether AMR was acquired at the community or nosocomially. Having isolates at admission, the point at which treatment decisions are also made would be able to answer this question of the timing of AMR acquisition.

## Conclusion

This study detected multiple PMQR genes and the first report of the *qepA* gene among CIP non-susceptible clinical *E. coli* and *Klebsiella* spp. The study observed high levels of CIP non-susceptibility and fluoroquinolone resistance carriage which could form a reservoir for the community spread of resistance, thus posing a great challenge in the effective treatment during hospital stays and subsequently during the post-hospital discharge period. We recommend enhanced surveillance for fluoroquinolone resistance carriage which will be vital to inform interventions to control antimicrobial-resistant bacteria and antimicrobial stewardship in rural and peri-urban populations.

## Materials and methods

### Study design

This was a cross-sectional nested study from the Toto Bora trial [44] that utilized *E. coli* and *Klebsiella* spp isolates recovered from fecal samples of children under five years discharged from two hospitals in western Kenya. Children being discharged from KTRH and HCRH aged between 1 -59 months were recruited in the parent study [44] to assess the effects of Azithromycin on mortality and rehospitalization in children under five years. The nested study used clinical, sociodemographic, and health history information collected during physical examination from children enrolled in the parent trial or interviews with their caregivers at hospital discharge. Faecal or rectal swab samples were collected before Azithromycin administration was done. The swabs were cultured, isolates recovered and biochemically identified as previously described [44].

### Parent trial

#### Bacterial isolation, identification, and AST

After laboratory culture, *E. coli* and *Klebsiella* spp isolates were identified and AST was performed by disc diffusion as previously described [45]. Briefly, a rectal swab or whole stool was collected from the enrolled child and was inoculated on MacConkey Agar and incubated at 37 °C for 24 h within 24 h of specimen collection time. Mucoid, lactose, or non-lactose fermenting colonies suspected to be *E. coli* or *Klebsiella* spp were isolated and the API 20E (bioMérieux, Inc, Durham, NC, United States) system confirmed the species of bacteria. A total of 568 children were randomly selected in the parent study, from whom 406 *E. coli* and 245 *Klebsiella* spp isolates were recovered and selected to have AST performed. The isolates were subjected to AST by disc diffusion to 5 μg of CIP (Oxoid, Hampshire, England) representing the fluoroquinolone antibiotic class. The isolates were also screened for ESBL production by the combined disc diffusion test and interpreted using the criteria from the Clinical and Laboratory Standards Institute (CLSI) [16]*. E. coli* ATCC 25922 and *E. coli* NCTC 13351 were used as negative and positive controls respectively for ESBL screening. *E. coli* or *Klebsiella* spp isolates with intermediate (22-25 mm) or resistant (≤ 21 mm) phenotype zone size interpretations for CIP were considered CIP non-susceptible [16].

### Nested study

#### CIP MIC determination by E-test

In the nested study, MICs for CIP were determined by the E-test method on isolates that were CIP non-susceptible by disc diffusion per CLSI guidelines [16]. Bacterial colonies were suspended in 0.85% normal saline to a turbidity equivalent to 0.5 McFarland standard (bioMérieux, Inc, Durham, NC, United States). The bacterial suspension was inoculated on Mueller Hinton agar (Oxoid, Hants, United Kingdom) plates, and the CIP E-strips were placed at the center of the agar followed by incubation at 35 °C for 16 -18 h. Concentration ranges for MICs for E-test strips for CIP (0.002–32 µg/mL) (bioMérieux Marcy l’Etoile, France) and non-susceptibility interpreted according to 2021 CLSI guidelines [16]. MIC results were classified as follows: susceptible (≤ 0.25 µg/mL), intermediate (0.5 µg/mL), or resistant (≥ 1 µg/mL). *E. coli* ATCC 25922 was used as quality control for determining MICs by the E-test method.

### DNA extraction and PMQR characterization

Genomic DNA was extracted using the boiling preparation method [46]. Extracted DNA was subjected to a series of single and multiplex PCR reactions to identify PMQR determinants: *qnrA, qnrB, qnrS*, *aac(6')-Ib*, *qepA, oqxA,* and *oqxB.* PCR reactions were performed using previously described primers and PCR conditions [47–49] (Table S2). All isolates positive for the *aac (6’)-lb* gene were further analyzed to determine carriage of the (-cr) variant associated with CIP resistance [48]. The PCR products and known positive strains were digested with the restriction enzyme *Bst*CI (New England Biolabs, Ipswich, MA) to identify *aac (6’)-lb-cr* which lacks the *Bst*CI restriction site present in CIP susceptible isolates as previously described [48]. The positive controls used in screening for PMQR genes were in-house isolates with confirmed target genes by whole genome sequencing and sequence analysis [32]. The identity of the amplified *qepA* gene was confirmed by amplicon sequencing of both the forward and reverse strands. PCR products positive for the *qepA* gene were purified using DNA Clean & Concentrator™-25 Kit (Zymo Research, Orange, CA, USA) and sequenced by the Sanger sequencing method using an ABI 3730 DNA analyzer. The consensus nucleotide sequences were analyzed and compared to available sequences deposited in the GenBank at National Center for Biotechnology Information (NCBI) using the Basic Local Alignment Search Tool (BLAST) program (http://blast.ncbi.nlm.nih.gov). The sequences of the *qepA* gene were submitted to GenBank.

### Statistical analysis

Fluoroquinolone resistance was defined by combining resistant and intermediate interpretative breakpoints for CIP. Risk factors for fluoroquinolone resistance previously associated with AMR from published data were chosen to test for association [50]. Patient characteristics including age, sex, hospital site, duration of hospitalization, antibiotic use at admission, HIV status, diagnosis at admission, and ESBL carriage were assessed. Poisson regression was used to determine prevalence ratios (PRs) and associated 95% confidence intervals (CIs) while Chi-square test was used to determine *p*-values. Associations were considered statistically significant at an alpha of 0.05. Analysis was performed in R software version 4.1.3.

### Supplementary Information

**Additional file 1:** **Table S1. **Correlates of non-susceptibility to Ciprofloxacin.

**Additional file 2:** **Table S2. **List of the PCR primer pairs and expected PCR product sizes.

## Data Availability

The datasets used and/or analyzed during the current study will be available from the corresponding author ppav@uw.edu. The *qepA* gene sequences from 8 representative isolates have been submitted to GenBank (https://www.ncbi.nlm.nih.gov/genbank/) under assigned accession numbers: OP918677 (E66), OQ031499 (E49), OQ031500 (E79), OQ031501 (E80), OQ031502 (E24), OQ031503 (E28), OQ031504 (E40), and OQ031505 (E42).

## Abbreviations

AMR: Antimicrobial Resistance
AST: Antimicrobial Susceptibility Testing
BLAST: Basic Local Alignment Search Tool
CIP: Ciprofloxacin
CLSI: Clinical and Laboratory Standard Institute
CMR: Centre for Microbiology Research
DNA: Deoxyribonucleic Acid
ESBL: Extended Spectrum Beta Lactamase
HCRH: Homabay County Referral Hospital
HIV: Human Immunodeficiency Virus
IQR: Interquantile range
KEMRI: Kenya Medical Research Institute
KTRH: Kisii Teaching and Referral Hospital
MGE: Mobile Genetic Elements
MIC: Minimum Inhibitory Concentration
NCBI: National Center for Biotechnology Information
PCR: Polymerase chain reaction
PMQR: Plasmid Mediated Quinolone Resistance
QRDR: Quinolone Resistance Determining Region
SSA: Sub-Saharan Africa
WHO: World Health Organization

## References

[1] Centers for Disease Control. Antibiotic resistance threats in the United States. Epub ahead of print 2019. 10.15620/cdc:82532.

[2] O’Neill J. Tackling drug-resistant infections globally: final report and recommendations: the review on antimicrobial resistance; 2016 Available from: https://amr-review.org. Publ html 2016; 1–35.

[3] Ramay BM, Caudell MA, Cordón-Rosales C, et al. Antibiotic use and hygiene interact to influence the distribution of antimicrobial-resistant bacteria in low-income communities in Guatemala. Sci Rep; 10. Epub ahead of print 1 December 2020. 10.1038/S41598-020-70741-4. 10.1038/s41598-020-70741-4 PMC7426860 32792543

[4] Kariuki S, Dougan G (2014). Antibacterial resistance in sub-Saharan Africa: an underestimated emergency. Ann N Y Acad Sci.

[5] Murray CJ, Shunji Ikuta K, Sharara F (2022). Global burden of bacterial antimicrobial resistance in 2019: a systematic analysis. Lancet.

[6] Tawfick MM, Elshamy AA, Mohamed KT (2022). Gut Commensal Escherichia coli, a High-Risk Reservoir of Transferable Plasmid-Mediated Antimicrobial Resistance Traits. Infect Drug Resist.

[7] Pilmis B, Le Monnier A, Zahar JR (2020). Gut Microbiota, Antibiotic Therapy and Antimicrobial Resistance: A Narrative Review. Microorg.

[8] Wallace MJ, Fishbein SRS, Dantas G. Antimicrobial resistance in enteric bacteria: current state and next-generation solutions. Gut Microbes; 12. Epub ahead of print 9 November 2020. 10.1080/19490976.2020.1799654. 10.1080/19490976.2020.1799654 PMC7524338 32772817

[9] Lamberte LE, van Schaik W (2022). Antibiotic resistance in the commensal human gut microbiota. Curr Opin Microbiol.

[10] WHO. Antimicrobial resistance. Global report on surveillance. Geneva PP - Geneva: World Health Organization. Epub ahead of print 2014. 10.1007/s13312-014-0374-3.

[11] Bruzzese E, Giannattasio A, Guarino A (2018). Antibiotic treatment of acute gastroenteritis in children. F1000Research.

[12] Cuypers WL, Jacobs J, Wong V, et al. Fluoroquinolone resistance in Salmonella: insights by whole-genome sequencing. Microb genomics; 4. Epub ahead of print 2018. 10.1099/mgen.0.000195. 10.1099/mgen.0.000195 PMC6113872 29975627

[13] Hooper DC, Jacoby GA (2015). Mechanisms of drug resistance: quinolone resistance. Ann N Y Acad Sci.

[14] Rodríguez-Martínez JM, Machuca J, Cano ME (2016). Plasmid-mediated quinolone resistance: Two decades on. Drug Resist Updat.

[15] Ruiz J. Transferable mechanisms of quinolone resistance from 1998 onward. Clin Microbiol Rev; 32. Epub ahead of print 1 October 2019. 10.1128/CMR.00007-19. 10.1128/CMR.00007-19 PMC6730498 31413045

[16] CLSI (2020). Performance Standards for Antimicrobial Susceptibility. Clsi.

[17] Parry CM, Vinh H, Chinh NT (2011). The Influence of Reduced Susceptibility to Fluoroquinolones in Salmonella enterica Serovar Typhi on the Clinical Response to Ofloxacin Therapy. PLoS Negl Trop Dis.

[18] WHO. Background document: The diagnosis, treatment and prevention of typhoid fever: Communicable Disease Surveillance and Response. Vaccines Biol Dep 2003; 1–33.

[19] WHO. Guidelines for the control of Shigellosis, including epidemics due to Shigella dysenteriae type 1. Geneva.

[20] Vien LTM, Minh NNQ, Thuong TC (2012). The Co-Selection of Fluoroquinolone Resistance Genes in the Gut Flora of Vietnamese Children. PLoS ONE.

[21] Salah FD, Soubeiga ST, Ouattara AK (2019). Distribution of quinolone resistance gene (qnr) in ESBL-producing Escherichia coli and Klebsiella spp. in Lomé Togo. Antimicrob Resist Infect Control.

[22] Maina M, Mwaniki P, Odira E (2020). Antibiotic use in Kenyan public hospitals: Prevalence, appropriateness and link to guideline availability. Int J Infect Dis.

[23] Yang P, Chen Y, Jiang S (2020). Association between the rate of fluoroquinolones-resistant gram-negative bacteria and antibiotic consumption from China based on 145 tertiary hospitals data in 2014. BMC Infect Dis.

[24] MoH. Policy Guidelines for Management of Diarrhoea in Children Below Five Years in Kenya. Ministry of Health, Kenya 2018; 36: 102–136.

[25] Chung The H, Le SNH (2022). Dynamic of the human gut microbiome under infectious diarrhea. Curr Opin Microbiol.

[26] Musicha P, Feasey NA, Cain AK (2017). Genomic landscape of extended-spectrum β-lactamase resistance in Escherichia coli from an urban African setting. J Antimicrob Chemother.

[27] Mahamat OO, Lounnas M, Hide M (2019). High prevalence and characterization of extended-spectrum ß-lactamase producing Enterobacteriaceae in Chadian hospitals. BMC Infect Dis.

[28] Leski TA, Stockelman MG, Bangura U (2016). Prevalence of Quinolone Resistance in Enterobacteriaceae from Sierra Leone and the Detection of qnrB Pseudogenes and Modified LexA Binding Sites. Antimicrob Agents Chemother.

[29] Abd El-Aziz NK, Gharib AA (2015). Coexistence of plasmid-mediated quinolone resistance determinants and AmpC-Beta-Lactamases in Escherichia coli strains in Egypt. Cell Mol Biol.

[30] Adekanmbi AO, Usidamen S, Akinlabi OC (2022). Carriage of plasmid-mediated qnr determinants and quinolone efflux pump (qepA) by ciprofloxacin-resistant bacteria recovered from Urinary Tract Infection (UTI) samples. Bull Natl Res Cent.

[31] Juma B. Molecular characterization of fluoroquinolone resistance genes in isolates obtained from patients with diarrhea in Machakos District Hospital, Kenya. African J Pharmacol Ther; 5.

[32] Musila L, Kyany’a C, Maybank R (2021). Detection of diverse carbapenem and multidrug resistance genes and high-risk strain types among carbapenem non-susceptible clinical isolates of target gram-negative bacteria in Kenya. PLoS One.

[33] Taitt CR, Leski TA, Erwin DP (2017). Antimicrobial resistance of Klebsiella pneumoniae stool isolates circulating in Kenya. PLoS ONE.

[34] Tulloch L, Martin E, Uslan DZ (2017). Clinical Outcomes of Patients Receiving Fluoroquinolones to Treat Bacteremia Caused by Enterobacteriaceae Isolates Considered Intermediate or Resistant to These Agents According to the Recently Revised Susceptibility Testing Standards by the Clinical and Laboratory Standards Institute (CLSI). Open Forum Infect Dis.

[35] Berendes D, Knee J, Sumner T (2019). Gut carriage of antimicrobial resistance genes among young children in urban Maputo, Mozambique: Associations with enteric pathogen carriage and environmental risk factors. PLoS ONE.

[36] Hu YS, Shin S, Park YH (2017). Prevalence and mechanism of fluoroquinolone resistance in Escherichia coli isolated from swine feces in Korea. J Food Prot.

[37] Hamed SM, Aboshanab KMA, El-Mahallawy HA (2018). Plasmid-mediated quinolone resistance in gram-negative pathogens isolated from cancer patients in Egypt. Microb Drug Resist.

[38] Robicsek A, Strahilevitz J, Jacoby GA (2006). Fluoroquinolone-modifying enzyme: a new adaptation of a common aminoglycoside acetyltransferase. Nat Med.

[39] Wairimu CW, Odari EO, Makobe CK (2021). Antimicrobial Susceptibility and Genetic Basis of Resistance of Klebsiella spp Isolated from Diarrheic and Non-Diarrheic Children at Health Facilities in Mukuru Informal Settlement, Nairobi. Kenya Adv Microbiol.

[40] Rahman Z, Islam A, Rashid M (2017). Existence of a novel qepA variant in quinolone resistant Escherichia coli from aquatic habitats of Bangladesh. Gut Pathog.

[41] Inwezerua C, Mendonça N, Calhau V (2014). Occurrence of extended-spectrum beta-lactamases in human and bovine isolates of Escherichia coli from Oyo state. Nigeria J Infect Dev Ctries.

[42] Hu F-P, Xu X-G, Zhu D-M (2008). Coexistence of qnrB4 and qnrS1 in a clinical strain of Klebsiella pneu-moniae 1.

[43] Kotb DN, Mahdy WK, Mahmoud MS (2019). Impact of co-existence of PMQR genes and QRDR mutations on fluoroquinolones resistance in Enterobacteriaceae strains isolated from community and hospital acquired UTIs. BMC Infect Dis.

[44] Pavlinac PB, Singa BO, Tickell KD, et al. Azithromycin for the prevention of rehospitalisation and death among Kenyan children being discharged from hospital: a double-blind, placebo-controlled, randomised controlled trial. Lancet Glob Heal; 0. Epub ahead of print September 2021. 10.1016/S2214-109X(21)00347-8. 10.1016/S2214-109X(21)00347-8 PMC8638697 34559992

[45] Tornberg-Belanger SN, Rwigi D, Mugo M (2022). Antimicrobial resistance including Extended Spectrum Beta Lactamases (ESBL) among E. coli isolated from kenyan children at hospital discharge. PLoS Negl Trop Dis.

[46] Cattoir V, Poirel L, Rotimi V (2007). Multiplex PCR for detection of plasmid-mediated quinolone resistance qnr genes in ESBL-producing enterobacterial isolates. J Antimicrob Chemother.

[47] Hong BK, Wang M, Chi HP (2009). oqxAB encoding a multidrug efflux pump in human clinical isolates of Enterobacteriaceae. Antimicrob Agents Chemother.

[48] Park CH, Robicsek A, Jacoby GA (2006). Prevalence in the United States of aac(6’)-Ib-cr encoding a ciprofloxacin-modifying enzyme. Antimicrob Agents Chemother.

[49] Robicsek A, Strahilevitz J, Sahm DF (2006). qnr prevalence in ceftazidime-resistant Enterobacteriaceae isolates from the United States. Antimicrob Agents Chemother.

[50] Katale BZ, Misinzo G, Mshana SE, et al. Genetic diversity and risk factors for the transmission of antimicrobial resistance across human, animals and environmental compartments in East Africa: A review. Antimicrob Resist Infect Control; 9. Epub ahead of print 6 August 2020. 10.1186/S13756-020-00786-7. 10.1186/s13756-020-00786-7 PMC7409632 32762743

