# Supplementary material for: Plasmid-mediated quinolone resistance genes detected in Ciprofloxacin non-susceptible Escherichia coli and Klebsiella isolated from children under five years at hospital discharge, Kenya
Source: BMC Microbiol. 2023 May 13;23:129. doi: 10.1186/s12866-023-02849-2 (PMC10182689; doi:10.1186/s12866-023-02849-2)
Supplement: Supplementary file 1 — Additional file 1: Table S1. Correlates of non-susceptibility to Ciprofloxacin. [file 12866_2023_2849_MOESM1_ESM.pdf]

Table S1: Correlates of non-susceptibility to Ciprofloxacin

	E. coli				*Klebsiella* spp			
	**Resistant**	**Susceptible**	**PR (95% CI)**	**P-value**	**Resistant**	**Susceptible**	**PR (95% CI)**	**P-value**
n	**N:188**	**N:218**	**	**	**N:92**	**N:153**	**	**
**Site**								
Homa Bay	78	86	**	**	35	63	**	**
Kisii	110	132	0.96 (0.77, 1.18)	0.68	57	90	1.09 (0.78, 1.52)	0.63
**Sociodemographic**								
**Age(months)**								
<6	23	21	**	**	13	26	**	**
≥6	165	197	0.87 (0.64, 1.18)	0.4	79	127	1.15 (0.71, 1.85)	0.55
**Age(months)**								
0-5	23	21	**	**	13	26	**	**
6-11	36	44	0.86 (0.59, 1.25)	0.44	20	38	1.03 (0.59, 1.83)	0.91
12-23	50	72	0.78 (0.55, 1.12)	0.2	26	37	1.24 (0.73, 2.11)	0.42
24-59	79	81	0.94 (0.68, 1.3)	0.73	33	52	1.16 (0.69, 1.95)	0.56
**Sex of child**								
Male	108	133	**	**	53	88	**	**
Female	80	85	1.08 (0.88, 1.34)	0.47	39	65	1 (0.72, 1.38)	0.99
**Duration of hospitalization (days)**								
0-2	53	75	**	**	15	39	**	**
3-4	57	86	0.96 (0.72, 1.28)	0.8	32	48	1.44 (0.87, 2.39)	0.15
>4	75	56	**1.38** (1.07, 1.78)	**0.01**	43	65	1.43 (0.72, 1.78)	0.13
**HIV status**								
HIV unexposed	167	173	**	**	75	128	**	**
HIV-exposed, uninfected	12	35	0.52 (0.32, 0.86)	**0.01**	14	19	1.15 (0.74, 1.78)	0.55
HIV infected	5	3	1.27 (0.74, 2.2)	0.45	2	4	0.9 (0.29, 2.84)	0.86
**Antibiotics used during admission (enrollment visit)**								
**Any antibiotics used?**								
No	15	37	**	**	4	22	**	**
Yes	173	181	**1.69** (1.09, 2.63)	**0.01**	88	131	**2.61** (1.05, 6.53)	**0.01**
**Ciprofloxacin**								
No	187	218	**	**	91	153	**	**

	E. coli				*Klebsiella* spp			
	**Resistant**	**Susceptible**	**PR (95% CI)**	**P-value**	**Resistant**	**Susceptible**	**PR (95% CI)**	**P-value**
Yes	1	0	**2.17** (1.95, 2.41)	**0.01**	1	0	**2.68** (2.28, 3.16)	**0.01**
**Penicillin**								
No	15	37	**	**	4	22	**	**
Yes	105	142	1.47 (0.94, 2.31)	0.09	62	106	2.40 (0.95, 6.04)	0.06
**Gentamicin**								
No	95	92	**	**	42	60	**	**
Yes	93	126	0.84 (0.68, 1.03)	0.09	50	93	0.85 (0.62, 1.17)	0.32
**Ceftriaxone**								
No	101	174	**	**	50	118	**	**
Yes	87	44	**1.81** (1.48, 2.2)	**0.01**	42	35	**1.83** (1.35, 2.5)	**0.01**
**Diagnosis on Admission**								
**Gastroenteritis/Diarrhoea**								
No	148	157	**	**	73	119	**	**
Yes	29	53	0.73 (0.53, 1.0)	**0.03**	15	28	0.92 (0.59, 1.43)	0.7
**Anemia**								
No	147	176	**	**	76	129	**	**
Yes	30	34	1.03 (0.77, 1.37)	0.84	12	18	1.08 (0.67, 1.73)	0.76
**Malaria**								
No	129	146	**	**	60	107	**	**
Yes	48	64	0.91 (0.71, 1.17)	0.47	28	40	1.15 (0.81, 1.62)	0.45
**Pneumonia**								
No	115	153	**	**	64	98	**	**
Yes	62	57	1.21 (0.97, 1.51)	0.09	24	49	0.83 (0.57, 1.22)	0.33
**Upper respiratory tract infection**								
No	164	191	**	**	80	138	**	**
Yes	13	19	0.88 (0.57, 1.36)	0.54	8	9	1.28 (0.75, 2.19)	0.4
**Malnutrition**								
No	168	189	**	**	82	134	**	**
Yes	9	21	0.64 (0.37, 1.11)	0.07	6	13	0.83 (0.42, 1.65)	0.58
**ESBL status**								
Negative	48	177	**	**	4	87	**	**
Positive	140	41	**3.63** (2.79, 4.72)	**0.00**	88	66	**13** (4.94, 34.22)	**0.00**

CI= Confidence interval; ESBL = Extented- spectrum beta-lacatamase; HIV= Human immunodeficiency virus; N= Number; PR= Prevalaence ratio
