# Supplementary material for: Plasmid-mediated quinolone resistance genes detected in Ciprofloxacin non-susceptible Escherichia coli and Klebsiella isolated from children under five years at hospital discharge, Kenya
Source: BMC Microbiol. 2023 May 13;23:129. doi: 10.1186/s12866-023-02849-2 (PMC10182689; doi:10.1186/s12866-023-02849-2)
Supplement: Supplementary file 2 — Additional file 2: Table S2. List of the PCR primer pairs and expected PCR product sizes. [file 12866_2023_2849_MOESM2_ESM.pdf]

Additional file 2

Table S2: List of the PCR primer pairs and expected PCR product sizes.

Primer	Sequence (5’-3’)	Band size	Reference
qnrA	F: ATTTCTCACGCCAGGATTTG<br>R: GATCGGCAAAGGTTAGGTCA	516 bps	[55]
qnrB	F: GATCGTGAAAGCCAGAAAGG<br>R: ACGATGCCTGGTAGTTGTCC	469bps	[55]
qnrS	F: ACGACATTCGTCAACTGCAA<br>R: TAAATTGGCACCCTGTAGGC	417 bps	[55]
aac(6')-Ib	F: TTGCGATGCTCTATGAGTGGCTA<br>R: CTCGAATGCCTGGCGTGTTT	482 bps	[54]
qepA	F: AACTGCTTGAGCCCGTAGAT<br>R: GTCTACGCCATGGACCTCAC	596 bps	[54]
oqxA	F: AACCTCGTCTCCCGTGAAGAGTG<br>R: TGAACGCTCTCCACCGCTTCAA	392 bps	[53]
oqxB	F:CAGCTCAACAATAAGGATGCGGTC<br>R:GGAGATCAGGAAATCGCTCTCCTG	512 bps	[53]

References

[53] Hong BK, Wang M, Chi HP, et al. oqxAB encoding a multidrug efflux pump in human clinical isolates of Enterobacteriaceae. *Antimicrob Agents Chemother* 2009; 53: 3582–3584.

[54] Park CH, Robicsek A, Jacoby GA, et al. Prevalence in the United States of aac(6’)-Ib-cr encoding a ciprofloxacin-modifying enzyme. *Antimicrob Agents Chemother* 2006; 50: 3953–5.

[55] Robicsek A, Strahilevitz J, Sahm DF, et al. qnr prevalence in ceftazidime-resistant Enterobacteriaceae isolates from the United States. *Antimicrob Agents Chemother* 2006; 50: 2872–2874.
